# Effects of an active break and postural shift intervention on preventing neck and low-back pain among high-risk office workers: a 3-arm cluster-randomized controlled trial

**DOI:** 10.5271/sjweh.3949

**Published:** 2021-04-27

**Authors:** Pooriput Waongenngarm, Allard J van der Beek, Nipaporn Akkarakittichoke, Prawit Janwantanakul

**Affiliations:** Department of Physical Therapy, Faculty of Allied Health Sciences, Chulalongkorn University, Bangkok, Thailand; Amsterdam UMC, Vrije Universiteit Amsterdam, Department of Public and Occupational Health, Amsterdam Public Health Research Institute, Amsterdam, The Netherlands

**Keywords:** computer, musculoskeletal disorder, posture, RCT, sedentary worker

## Abstract

**Objective:**

This study evaluated the effects of the promotion of active breaks and postural shifts on new onset of neck and low-back pain during a 6-month follow-up among high-risk office workers.

**Methods:**

A 3-arm cluster-randomized controlled trial with 6-month follow-up was conducted among healthy but high-risk office workers. Participants were recruited from six organizations in Bangkok, Thailand (N=193) and randomly assigned at cluster level into active break intervention (N=47), postural shift intervention (N=46), and control (N=100) groups. Participants in the intervention groups received a custom-designed apparatus to facilitate designated active breaks and postural shifts during work. Participants in the control group received a placebo seat pad. The primary outcome measure was new onset of neck and low-back pain during 6-month follow-up. Analyses were performed using Cox proportional hazard models.

**Results:**

One-hundred and eighty-six (96%) predominantly female participants were successfully followed up over six months. New onset of neck pain during the 6-month follow-up occurred in 17%, 17%, and 44% of the participants in the active break, postural shift, and control groups, respectively. For new onset of low-back pain, these percentages were 9%, 7%, and 33%, respectively. Hazard rate (HR) ratios after adjusting for biopsychosocial factors indicated a protective effect of the active break and postural shift interventions for neck pain [HR_adj_ 0.45, 95% confidence interval (CI) 0.20–0.98 for active break and HR_adj_ 0.41, 95% CI 0.18–0.94 for postural shift] and low-back pain (HR_adj_ 0.34, 95% CI 0.12–0.98 for active break and HR_adj_ 0.19, 95% CI 0.06–0.66 for postural shift).

**Conclusion:**

Interventions to increase either active breaks or postural shifts reduced new onset of neck and low-back pain among high-risk office workers.

Neck and low-back pain are major health problems for office workers. Neck pain is prevalent among office workers. For example, 46% of office workers in Iran reported neck pain in the past year (1) and 31% of office workers in Thailand developed a new episode of neck pain in the previous year (2). Low-back pain affected 51% of office workers in Nigeria annually (3), while 14% of office workers in Thailand reported new onset of low-back pain in the past year (4). Neck and low-back pain are often the cause of significant physical and psychological health impairments, which affect work performance and social responsibilities (5, 6). Consequently, neck low-back pain constitute a great socioeconomic burden on both individuals and society as a whole (6, 7).

Office work mainly involves computer use, participation in meetings, reading, and phoning. A typical workday for many office workers is characterized by desk-based work, which entails several hours of sitting. Individuals with prolonged sitting have been found to experience increased musculoskeletal discomfort over time, particularly in the neck and low back (8, 9). Evidence suggests that signs of bodily perceived discomfort, such as tension, fatigue, soreness, or tremors, are predictors of musculoskeletal disorders (10).

A number of interventions have been proposed to alleviate the adverse effects of prolonged sitting, including breaks (11–13), postural shifts (14, 15), and ergonomic intervention (16). A recent systematic review showed a positive effect of rest breaks with postural change or active breaks on pain and discomfort (11). Postural shifts while sitting, defined as body movements causing significant changes in the load on the left and right ischial tuberosities for the sagittal and frontal planes (15), are regarded as a natural coping response to diminish the perception of discomfort and relieve the perceived pressure of compressed body parts (17). Previous research has found similar trends linking increased motion with decreased discomfort in the low back during prolonged sitting (18, 19). Thus, promotion of rest breaks and postural shifts during sitting may be an effective intervention in the reduction of neck and low-back pain.

To the best of our knowledge, there has been no randomized trial investigating the efficacy of rest break and postural shift interventions in the prevention of neck and low-back pain among office workers. Therefore, the aim of this study was to evaluate the effect of the promotion of rest breaks and postural shifts on new onset of neck and low-back pain during 6-month follow-up among high-risk office workers. We hypothesized that participants in the intervention groups, with increases in either rest breaks or postural shifts, show reduced new onset of neck and low-back pain.

## Methods

### Participants

A 3-arm, parallel-group, cluster-randomized controlled trial with 6-month follow-up was conducted in a convenience sample of office workers recruited from six organizations in Bangkok, Thailand. The organizations participating in this study were the government excise, public relations, and public transportation departments, the Metropolitan Waterworks Authority, and two private companies importing medical equipment and products (such as drugs and diagnostic reagents). Individuals were included in the study if they: were aged 23–55 years, worked full-time, had a body mass index (BMI) of 18.5–25 kg/m^2^, had ≥5 years of experience in their current position, and were at risk of non-specific neck pain as evaluated by the Neck Pain Risk Score for Office Workers (NROW; score ≥2) (20) and non-specific low-back pain as evaluated by Back Pain Risk Score for Office Workers (BROW; score ≥53) (21). Participants were excluded if they had reported musculoskeletal symptoms in the neck or low back in the previous six months, reported pregnancy or had planned to become pregnant in the coming 12 months, had a history of trauma or accidents in the spinal region, or had either spinal, intra-abdominal or femoral surgery in the previous 12 months. Participants who had been diagnosed with congenital anomaly of the spine, rheumatoid arthritis, infections of the spine or discs, ankylosing spondylitis, spondylolisthesis, spondylosis, spinal tumor, systemic lupus erythymatosus, or osteoporosis were also excluded from the study.

Office workers were invited to participate in this study and those who expressed interest completed a short screening questionnaire, assessing aforementioned inclusion and exclusion criteria using the NROW and BROW. The NROW comprises three questions concerning lifetime history of neck pain, chair adjustability, and perceived muscular tension. The NROW has scores of 0–4. A cut-off score of ≥2 had a sensitivity of 82% and specificity of 48%. The positive and negative predictive values were 29% and 91%, respectively. The BROW consists of two questions concerning lifetime history of low-back pain and psychological demands. The BROW has scores of 12–69. With a cut-off score of 53, the sensitivity was 65% and the specificity was 68%. The positive and negative predictive values were 16% and 95%, respectively. If eligible, potential participants were informed about the objectives and details of the study and asked to provide informed consent to participate in the research.

At baseline, participants completed the self-administered questionnaire for exposure data, ie, confounders. Participants were assigned at cluster level into either the intervention A (active break), intervention B (postural shift), or control groups by a simple randomization method. A researcher with no other involvement in the trial prepared the designation of intervention by using computer-generated randomization. Both data collectors and the analyst were not involved in the group assignment process. Clusters of participants were located in the same workplace to avoid contamination of the intervention and enhance compliance within the intervention group (22). A total of six clusters (two clusters for the intervention group A, two clusters for the intervention group B, and two clusters for the control group) were identified and cluster size range was 15–51 participants. Participants then received a self-administered diary to record any new onset of neck or low-back pain and, if occurring, its intensity and any resulting disability. The researcher collected the diaries from participants every month over a 6-month period. The University Human Ethics Committee approved the study, which was registered in the Thai Clinical Trials Registry (TCTR20190111002). No changes had been made to the methods after trial commencement until March 2020, when the COVID-19 outbreak occurred in Thailand. At the time, a majority of the participants (68%) in this study were forced to work from home and did not bring the custom-designed apparatus home. Furthermore, a previous study reported no relationship between the prevalence of neck and low-back symptoms and the seasons (23).

### Baseline questionnaires

The Borg CR-10 scale was used to determine perceived discomfort (24). Participants were asked to indicate how much discomfort was felt in the past year in the neck and low back (on a 0–10 scale; 0 denotes no discomfort and 10 denotes extreme discomfort). Neck and low-back regions were defined according to a chart based on the modified Nordic questionnaire (25). In addition, the following biopsychosocial characteristics were obtained: individual, work-related (physical) factors and psychosocial work characteristics. Individual factors included gender, age, education level, frequency of regular exercise or sport, and smoking habits. Work-related (physical) factors included current job position, number of working hours, years of work experience, frequency of using a computer, adopting working postures, performing various work activities, and rest breaks. The questionnaire also asked respondents to self-rate (yes or no) the ergonomics of their workstations (whether the desk height was suitable for them, they used a height-adjustable chair, and the top of the computer screen was positioned at a level horizontal with their eyes) and work environment conditions (the appropriateness of ambient temperature, noise level, light intensity, and air circulation). Psychosocial work characteristics were measured using the Thai version of the Job Content Questionnaire (26). The questionnaire comprises 54 items in the following six areas: psychological demands (12 items), decision latitude (11 items), social support (8 items), physical demands (6 items), job security (5 items), and hazards at work (12 items). Each item has four Likert-type response options ranging from 1: strongly disagree, to 4: strongly agree, that were summarized to obtain a sum score per area.

### Description of intervention

Participants in the intervention A (active break) and intervention B (postural shift) groups received a custom-designed apparatus, which consisted of three components: (i) a seat pad (width × length × height = 40 cm × 50 cm × 1 cm), (ii) a processor, and (iii) a smartphone application. The seat pad was used to collect data regarding sitting behavior, including sitting and break duration as well as number of postural shifts. Data were stored in the processor, which were used to calculate recommended active breaks and postural shifts for each individual. Instructions to have active breaks were sent from the processor to the smartphone application via Bluetooth technology. Designated postural shifts were induced by the apparatus gradually pumping the air into various parts of the seat pad placed underneath a participant’s buttocks. Commands to operate the seat pad were sent from the processor to the seat pad via a cord connected between them. The apparatus was installed by the researcher at participants’ workplaces. The researcher explained and demonstrated how to use the apparatus and participants were asked to follow the instructions conveyed via the smartphone application, ie, having active breaks or postural shifts, as much as possible.

Each participant in the intervention A (active break) group was asked to have designated active breaks during the workday, and they were asked not to be seated in a chair when taking the breaks. The frequency and duration of breaks were based on the theoretical effects of rest breaks on the reduction of neck and low-back discomfort (11), ranging from 30 seconds to 15 minutes per break and 0–30 times per workday, depending on their occupational sitting behavior.

Each participant in the intervention B (postural shift) group was asked to make designated postural shifts during each workday. The frequency of postural shifts was based on the theoretical effects of postural shifts on the reduction of neck and low-back discomfort (15, 27), ranging from 20–60 times per hour, depending on their occupational sitting behavior. The occupational sitting behaviors of participants in both intervention groups during the trial were assessed using the aforementioned custom-designed apparatus and collected every month during follow-up.

Participants in the control group received a placebo seat pad made of polypropylene foam (width × length × height = 40 cm × 50 cm × 1 cm) to be placed on the seat pan of a chair. During the study, participants in all groups were asked to keep the level of their leisure time physical activity unchanged.

### Follow-up outcome measure

The new onset of non-specific neck or low-back pain – with or without radiation and without any specific systematic disease being detected as the underlying cause of the complaints (28, 29) – during the 6-month follow-up period was collected using a diary. Participants answered the yes/no question “Have you experienced any neck or low-back pain lasting >24 hours during the past month?”. If they answered “Yes”, follow-up questions about pain intensity measured by a visual analogue scale and the presence of weakness or numbness in the upper limbs were asked. Those who answered “Yes” to the first question, reported pain intensity >30 mm on a 100-mm visual analogue scale and had no weakness or numbness in the upper or lower limbs were identified as cases. Participants who reported new onset neck and low-back pain were also asked about their disability level as measured using the neck disability index (NDI) (30) or Roland-Morris low-back disability questionnaire (RMDQ) (31), respectively. The NDI contains 10 items on a 5-point Likert scale and the total score ranges from 0–50, with higher scores indicating more severe disability. The RMDQ comprises 24 items and the total score is the sum of the ticked boxes, ranging from 0–24, with higher scores indicating more severe disability. Participants were followed until they completed the 6-month follow-up or withdrew from the study.

### Statistical analysis

Comparisons of the baseline characteristics of participants between the intervention A (active break), intervention B (postural shift), and control groups were conducted using one-way ANOVA for continuous data and χ^2^ test for nominal and ordinal data. All analyses followed an intention-to-treat approach. Missing data were handled using the “hot-deck imputation” procedure. A respondent was selected at random from the total sample of the study, and the value for that person was assigned to the case for which information was missing. This procedure was conducted repeatedly for each missing value, until the dataset was complete. The 6-month incidence rate of neck and low-back pain was calculated for each group as the proportion of new cases reporting neck or low-back pain during the 6-month follow-up. Further follow-up data of those initially identified as cases were not used any further.

Survival analysis was used to determine Kaplan-Meier survival curves for the intervention A (active break), intervention B (postural shift), and control groups. Survival time was taken as the time (in months) from the start to the incident symptoms becoming manifested. Those participants who left the study without manifesting symptoms were no longer recorded at the time they left. The two survival curves generated by the Kaplan-Meier method were compared using the log rank test.

Hazard ratios (HR) with respect to incident cases for neck and low-back pain were calculated using the Cox proportional hazards model. Gender, age, and psychological scores were forced into all models to reduce confounding due to these factors. The other 40 possible covariates were each examined in multivariate models. If the tested covariate changed the HR of the intervention variable by ≥0.05 then it was also included in the final, adjusted model.

To determine the effects of intervention A (active break) and intervention B (postural shift) on neck and low-back discomfort scores during the 6-month follow-up period, the Borg CR-10 scores were analyzed using a two-way analysis of covariance (ANCOVA), using the Borg CR-10 score at baseline as covariate, with one within-subjects factor (time) and one between-subjects factor (group). When a significant interaction between time and group was detected, the effects of each variable was examined separately using one-way ANCOVA. The Bonferroni correction procedure was applied to determine where statistical significance occurred.

Health outcomes, ie, pain intensity and disability for those reporting neck and low-back pain, were compared between the intervention A (active break), intervention B (postural shift), and control groups using one-way ANOVA. All statistical analyses were performed using SPSS for Windows Version 23.0 (IBM, Armonk, NY, USA). Statistical significance was set at the 5% level.

## Results

**Figure 1:**
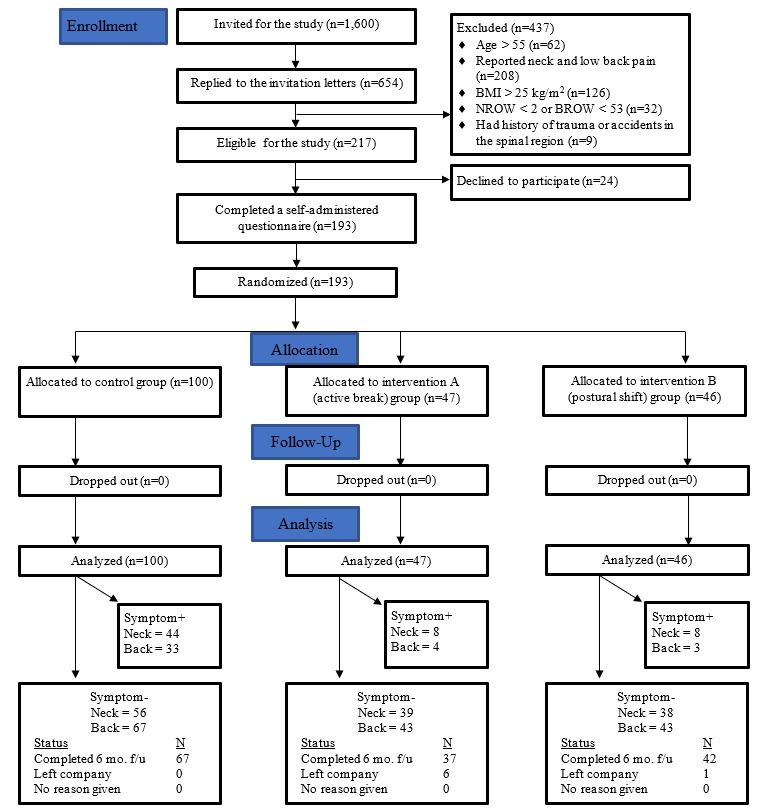
Consolidated Standards of Reporting Trials (CONSORT) flowchart of the study.

The trial ran from June 2019 to April 2020. Of the total 1600 workers who received the invitation, 654 responded (response rate: 40%). In total, 217 were eligible, 193 of whom agreed to participate in the study. Of those, 186 were successfully followed for six months and 7 (4%) were lost during the follow-up period because they left the organisations (figure 1). The sample population comprised mainly females (76%) (table 1). Their average age was 33.8 (6.3) years. Most of the participants (95%) had graduated with at least a bachelor’s degree. There were no significant differences in any of the characteristics of the participants among the three groups, except for age, BMI, education level, duration of employment, psychological job demand, and social support. All occupational sitting behaviors from participants in both intervention groups are presented in table 2.

**Table 1:** Baseline characteristics of participants. [I=intervention; SD=standard deviation.]

Characteristic	I–A (active break) (N=47)	I-B (postural shift) (N=46)	Control (N=100)	P-value
		
	Mean (SD)	Mean (SD)	Mean (SD)	
Demographic				
Age (years)	31.6 (6.1)	35.5 (7.7)	34.1 (5.3)	0.009 ^a^
Gender: female (%)	33 (70.2)	35 (76.1)	79 (79.0)	0.507
Body weight (kg)	57.3 (10.5)	60.2 (10.2)	56.4 (13.7)	0.208
Body height (cm)	163.0 (9.1)	162.9 (7.9)	161.4 (6.9)	0.376
Body mass index (kg/m^2^)	21.3 (2.3)	22.3 (2.3)	21.0 (2.0)	0.004 ^a^
Education (%)				0.001 ^a^
<Bachelor’s degree	2 (4.3)	2 (4.3)	5 (5.0)	
Bachelor’s degree	40 (85.1)	38 (82.6)	53 (53.0)	
>Bachelor’s degree	5 (10.6)	6 (13.1)	42 (42.0)	
Exercise frequency in the past 12 months (%)				0.204
Never	6 (12.8)	5 (10.9)	22 (22.0)	
Occasionally	34 (72.3)	30 (35.2)	56 (56.0)	
Regularly	7 (14.9)	10 (21.8)	22 (22.0)	
Not sure	0 (0.0)	1 (2.1)	0 (0.0)	
Work-related				
Employment (years)	6.9 (4.3)	10.8 (5.3)	9.1 (4.8)	0.001 ^a^
Working hours per day	8.0 (1.3)	8.7 (1.3)	7.8 (0.8)	0.068
Working days per week	5.1 (0.3)	4.8 (0.6)	5.0 (0.2)	0.052
Psychosocial				
Job control	35.1 (4.5)	35.0 (5.2)	36.6 (4.3)	0.070
Psychological job demands	30.8 (4.4)	32.5 (4.2)	33.2 (4.4)	0.009 ^a^
Physical job demands	13.2 (2.7)	13.4 (3.3)	14.1 (2.6)	0.120
Job security	16.3 (1.3)	16.3 (2.9)	16.9 (1.1)	0.073
Social support	33.1 (4.4)	30.4 (3.2)	32.9 (4.4)	0.001 ^a^
Hazards at work	15.9 (3.9)	15.5 (2.5)	17.0 (3.9)	0.051

a P-value <0.05.

**Table 2:** Occupational sitting behaviors. [I=intervention; SD=standard deviation.]

Variables	I–A (active break) group (N=47)	I–B (postural shift) group (N=46)
	
	Mean (SD)	Mean (SD)
Sitting duration at work per day (min)	295.8 (130.9)	263.2 (154.4)
Break duration per day (min)	85.4 (44.1)	
Average break duration (min)	3.1 (1.7)	
Number of breaks per day	32.5 (20.4)	
Number of total postural shifts		27.3 (7.4)

In March 2020, the COVID-19 outbreak occurred in Thailand, which forced a majority of the participants in the present study (68%) to work from home. At the time, we had completed the 6-month follow-up for the participants in the control and intervention A (active break) groups. However, the participants in the intervention B (postural shift) group were followed up for only the first 4 months. Thus, it should be noted that data from the 5^th^ and 6^th^ months of participants in the intervention B (postural shift) group were collected while they were working from home (during March to April 2020), and these months were used for statistical analyses in this study, following the intention-to-treat principle. All participants reported that they did not bring the custom-designed apparatus for use at home.

To investigate the effect of working from home in the intervention B (postural shift) group, we compared the 6-month follow-up results to those of 4-month follow-up (ie, excluding the last 2 months). No alteration of the findings was found between the two sets of data (results not shown). The 6-month follow-up results are given below.

### New onset of neck and low-back pain

Over the 6-month follow-up, 17% (8/47) of participants in the intervention A (active break), 17% (8/46) of those in the intervention B (postural shift), and 44% (44/100) of those in the control group reported onset of neck pain. For low-back pain, 9% (4/47) of participants in intervention A (active break), 7% (3/46) of those in intervention B (postural shift), and 33% (33/100) of those in the control group reported onset of low-back pain. No harmful or unintended effects were reported among the participants in the three groups.

**Figure 2:**
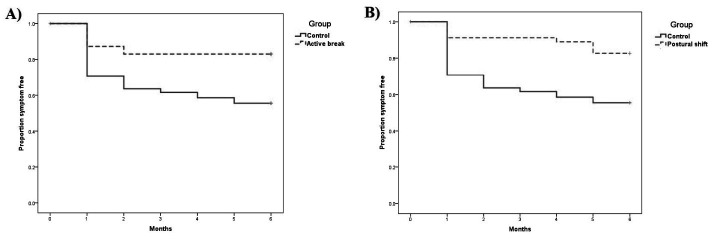
The Kaplan–Meier survival curves for onset of neck pain: A) Intervention A (active break) and B) Intervention B (postural shift).

**Figure 3:**
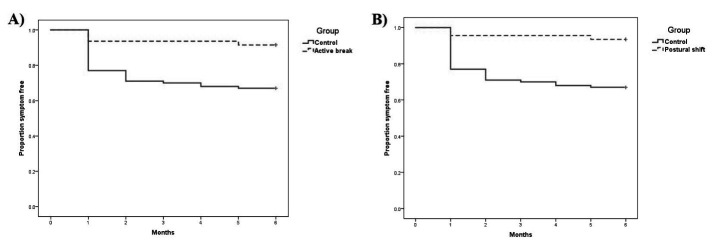
The Kaplan–Meier survival curves for onset of low-back pain: A) Intervention A (active break) and B) Intervention B (postural shift).

The Kaplan–Meier survival curves for the neck and low-back cohort illustrated a significant difference in time to neck and low-back pain between the intervention A (active break) and control group (log rank test probability = 0.002), and the intervention B (postural shift) and control group (log rank test probability = 0.001) (figures 2 and 3). Participants in the control group had greater risk of neck and low-back pain than those in the intervention A (active break) and intervention B (postural shift) groups.

Using the Cox proportional hazard model, after adjustment for age, gender, education level, duration of employment, seat height, and psychosocial work characteristics, the protective effects of intervention A (active break) and intervention B (postural shift) were found for neck and low-back pain. Intervention A (active break) significantly reduced the risk of incident neck pain [HR_adj_ 0.45, 95% confidence interval (CI) 0.20–0.98, P=0.047] and low-back pain (HR_adj_ 0.34, 95% CI 0.12–0.98, P=0.047). Intervention B (postural shift) significantly reduced the risk of incident neck pain (HR_adj_ 0.41, 95% CI 0.18–0.94, P=0.035) and low-back pain (HR_adj_ 0.19, 95% CI 0.06–0.66, P=0.009) (table 3). Comparisons of pain intensity and disability level among the intervention A (active break), intervention B (postural shift), and control groups indicated no statistically significant difference (table 4).

**Table 3:** Unadjusted and adjusted hazard rates (HR) evaluating the effects of intervention A (I–A: active break) and intervention B (I–B: postural shift) on incident neck and low-back pain (N=193). [CI=confidence interval.]

	Incidence (%)	Unadjusted	P-value ^a^	Adjusted ^b^	P-value ^a^
	
		HR (95% CI)		HR (95% CI)	
Neck pain					
Control group (N=100)	44 (44)	1.00		1.00	
I–A (active break) group (N=47)	8 (17)	0.36 (0.17–0.75)	0.007	0.45 (0.20–0.98)	0.047
I–B (postural shift) group (N=46)	8 (17)	0.35 (0.16–0.74)	0.006	0.41 (0.18–0.94)	0.035
Back pain					
Control group (N=100)	33 (33)	1.00		1.00	
I–A (active break) group (N=47)	4 (9)	0.24 (0.08–0.67)	0.007	0.34 (0.12–0.98)	0.047
I–B (postural shift) group (N=46)	3 (7)	0.18 (0.06–0.59)	0.005	0.19 (0.06–0.66)	0.009

a P-value <0.05.

b Variables; age, gender, education level, duration of employment, seat height, job control, psychological job demand, physical job demand, job security, social support, hazards at work, and neck/low-back discomfort.

**Table 4:** Pain intensity and disability of participants reporting neck and low-back pain during 6-month follow-up. [I=intervention; SD=standard deviation; VAS=visual analogue scale; NDI=neck disability index; RMDQ=Roland-Morris low-back disability questionnaire.]

Variables	I–A (active break) group		I–B (postural shift) group		Control group		P-value
		
	Mean (SD)	N	Mean (SD)	N	Mean (SD)	N	
Neck pain							
Pain intensity measured by VAS	3.4 (0.4)	8	5.2 (1.7)	8	4.0 (1.6)	44	0.070
Disability measured by NDI	7.4 (2.8)	8	6.0 (3.5)	8	3.9 (0.6)	44	0.761
Back pain							
Pain intensity measured by VAS	4.0 (1.4)	4	3.0 (0.5)	3	3.8 (1.9)	33	0.725
Disability measured by RMDQ	2.7 (1.5)	4	2.0 (0.0)	3	1.9 (1.5)	33	0.548

**Figure 4:**
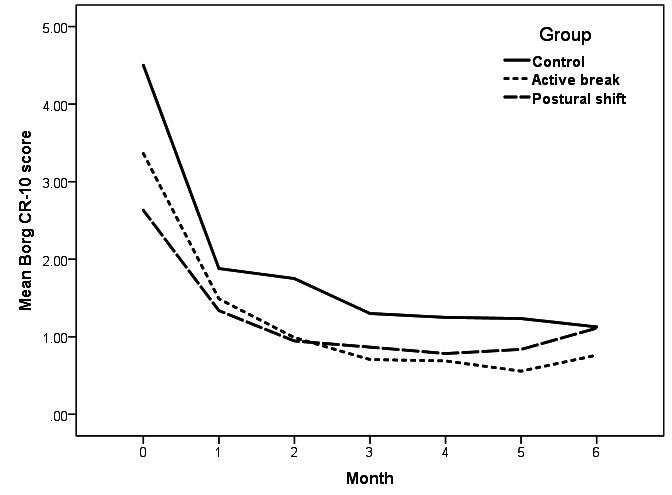
Mean Borg CR-10 scores at the neck over the 6-month follow-up period for intervention A (active break) (N=47), intervention B (postural shift) (N=46), and control groups (N=100).

**Figure 5:**
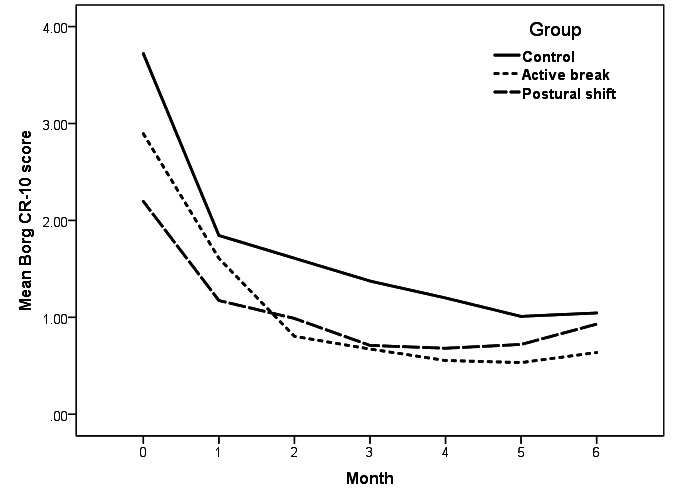
Mean Borg CR-10 scores at the low back over the 6-month follow-up period for intervention A (active break) (N=47), intervention B (postural shift) (N=46), and control groups (N=100).

A two-way ANCOVA, with Borg CR-10 score at baseline as covariate, indicated significant effects for time (F_5,825_=2.769, P=0.017), group (F_2,165_=2.319, P=0.102), and their interaction (F_10,825_=0.902, P=0.531) on neck discomfort score (figure 4). Also, there were significant effects of time (F_5,825_=3.591, P=0.003), group (F_2,165_=3.589, P=0.030) and their interaction (F_10,825_=1.012, P=0.431) on low-back discomfort score (figure 5). Thus, further analyses were performed.

The post-hoc Bonferroni test showed that neck and low-back discomfort scores after 3 and 2 months of all groups were significantly lower than those at baseline (P<0.05), respectively. Only a significant difference in low-back discomfort scores was found between the intervention A (active break) and control groups during 6-month follow-up (P<0.05). There was no significant difference in neck and low-back discomfort scores between the intervention B (postural shift) and control groups during 6-month follow-up (P>0.05).

## Discussion

This randomized controlled trial showed that the rest break and postural shift intervention delivered by the custom-designed apparatus reduced the new onset of neck and low-back pain during 6-month follow-up among high-risk office workers. The 6-month onset of neck and low-back pain was reduced by 55–81% by the interventions. However, neither the rest break nor the postural shift intervention reduced pain intensity or disability level in those experiencing neck and low-back pain.

In this study, the 6-month onset of neck and low-back pain in office workers of the control group were 44% and 33%, respectively. These findings are in line with a previous study by Sitthipornvorakul et al (32), showing the 6-month incidence of neck pain among office workers to be 34%. However, Lapointe et al (33) reported the 6-month onset of neck and low-back pain among office workers to be 18% and 14%, respectively. The discrepancy between our study and that of Lapointe et al (33) may be due to the difference in the inclusion criteria. Lapointe et al (33) did not require participants to be at risk of neck or low-back pain. However, in our study office workers at risk of neck and low-back pain, assessed by the NROW and BROW, were included. Consequently, it is plausible that a greater number of participants experienced neck and low-back pain over the course of our study. The high-risk study population also puts the present study’s relatively large effect sizes in perspective; it should be kept in mind that the majority of office workers (ie, those not at risk of neck and low-back pain as well as those who reported neck or low-back symptoms in the previous 6 months) were not included in the present study. Prevention targeted at a high-risk group is different from preventive efforts aimed at all employed office workers (34).

Sitthipornvorakul et al (32) has reported that a walking intervention can largely reduce the 6-month incidence rate of neck pain (adjusted odds ratio of 0.22) among high-risk healthy office workers, for which the same inclusion criteria as those in the present study were used. Danquah et al (35) also found a reduction in the prevalence of neck pain after their 3-month intervention among office workers, who received the Take a Stand! intervention aimed to reduce sitting time (adjusted odds ratio of 0.52). They found, however, no change in low-back pain. A systematic review and meta-analysis indicated that only exercise intervention was effective for reducing the occurrence of low-back pain (pooled risk ratio of 0.65) (36). However, other systematic reviews reported that rest breaks were an effective intervention to reduce pain and discomfort in various body regions (particularly in the low back), which is secondary prevention for musculoskeletal disorders (11, 37).

The present study found that active breaks can reduce new onset of neck and low-back pain by 55% and 66%, respectively. Our results showed that the average break duration of participants in the active break group was 3.1 minutes. Previous studies have found frequent active breaks with postural change, with break durations ranging from 20 seconds to 5 minutes, to be beneficial in reducing pain, discomfort, and fatigue in the neck and low back (12, 13, 38). The number of active breaks in the active break group of the present study was 32.5 times per workday and was higher than that reported by Renaud et al (39), who showed 28.3 sit-stand transitions per workday. The discrepancy between our and previous studies may be partly attributed to the use of the intervention apparatus. Scheduled rest breaks have been recommended to decrease musculoskeletal discomfort and pain during computer tasks (13, 40) and active breaks with postural change were found to be effective in reducing pain and discomfort (11). Active breaks with postural change require participants to change their posture during breaks, which may lead to improvement in blood circulation in the lumbar region, change in spinal curvature, delay in the onset of any specific musculoskeletal discomfort, and increase in the flow of synovial fluid to lubricate and nourish the intervertebral disc (41, 42). Changing posture when adopting prolonged, sustained, and awkward sitting postures may prevent a reduction in the length of soft tissues and range of motion in joints, which may reduce the risk of injury (43). Therefore, frequent active breaks of short duration may be sufficient to prevent the onset of neck and low-back pain among high-risk office workers. Future studies should evaluate the impact of frequent and short breaks on work productivity to determine the feasibility of implementing our break program in a real working life setting.

Our results indicated that the postural shifts intervention can prevent the onset of neck and low-back pain by 59% and 81%, respectively. The number of total postural shifts found in the postural shift group of the present study was 27.3 times per hour, which was much higher than those reported in previous studies (8–10 times per hour in a normal work situation) (15, 27). Again, the discrepancy in number of postural shifts between our and previous studies may be partly attributed to the use of the apparatus. Previous studies indicated that increased motion during prolonged sitting has been found to decrease discomfort in the neck and low back (44, 45). Postural shift has been shown to increase subcutaneous oxygen saturation on average by 2.2% with each posture adjustment, indicating the positive effects of posture shifts on tissue viability (15). Static neck posture is a possible risk factor in neck pain (46). A previous study found that individuals with low-back pain had less frequent postural shifts than their healthy counterparts (47). Changing sitting postures has been found to result in different levels of cervicothoracic muscle activity (48). Hence, changing sitting postures may impose alternating activity between different parts of the neck and shoulder muscles resulting in alleviated postural discomfort during prolonged sitting. Increased postural movement whilst sitting has been associated with less spinal load and reduced loss of disc height (14, 49). Thus, our results suggest that frequency of postural shifts may partly be related to the occurrence of neck and low-back pain in those required to sit for long periods and at increased risk of neck and low-back pain.

Our results showed that neck and low-back discomfort scores in all three groups significantly decreased within the first 2–3 months. One plausible explanation of such a finding relates to participant expectations, which has been established as a key process behind the placebo effect (50). A previous study showed that placebo appears to be effective with subjective outcomes (51). Neck and low-back discomfort scores in the intervention groups were lower than those in the control group during 6-month follow-up, although the differences did not reach statistical significance. It should be noted that neck and low-back discomfort scores of participants in the intervention B (postural shift) group increased moderately at the 5^th^ and 6^th^ months of follow-up. At the time, the COVID-19 outbreak occurred in Thailand and the participants in the intervention B (postural shift) group were forced to work from home and did not bring the custom-designed apparatus for use at home. The results support the notion that reduced new onset of neck and low-back pain among those receiving either active break or postural shift may emanate from a decrease in discomfort in the neck and low back.

In the present study, no significant differences were found in pain intensity or disability between the groups. These results support the notion that effective interventions to prevent neck and low-back pain, at least among office workers, may differ from those to alleviate pain intensity and disability level among those with neck and low-back pain. Disability levels due to neck or low-back pain among the present study population, ie, those who reported pain, were relatively low. Consequently, we may have encountered a floor effect, ie, participants scored at or near the possible lower limit (52). Further research should examine the effects of active break and postural shift intervention in office workers with moderate to high pain intensity or disability to validate the findings of the present study.

A major strength of this study is its randomized design and the inclusion of a broad range of psychosocial factors for their confounding effect on neck and low-back pain. Moreover, use of the placebo seat pad in the control group may have reduced the placebo or Hawthorne effect on the outcomes of this study. Four methodological limitations should be taken into consideration when interpreting the results of this study. First, the present study was conducted among healthy office workers with specific characteristics, including being 23–55 years of age, having ≥5 years of experience in the current position, having high risk of neck and low-back pain, and not presenting any of several medical conditions. Thus, extrapolation of these results to other populations should be made with caution. Further research on the effects of active break and postural shift intervention on the onset of neck and low-back pain in normal office worker populations or other occupations is suggested. Second, assessments of biopsychosocial factors as well as the diagnosis of neck and low-back pain were subjective, which poses the risk of bias in the estimation of exposure or health outcome. Researchers should consider the inclusion of objective information from physical examination to increase data accuracy in future studies. Third, the population in this study comprised mainly females and some baseline characteristics showed differences among the three study groups. Following the use of cluster randomization, participants were randomized as intact groups rather than as individuals. A small number of clusters (N=6) were randomized in this study, which had the risk of baseline imbalance between the randomized groups. Thus, further research should use stratified or pair-matched randomization of clusters (53). Last, we did not assess participants’ sitting behavior at baseline. Therefore, we did not know whether the designated active breaks and postural shifts suggested by the apparatus for individuals in the intervention A and B groups were higher or lower than their habitual daily occupational sitting behavior. Due to the limitation of the custom-designed apparatus, we did not assess the compliance of participants in the intervention groups during the follow-up period. It is plausible that, for example, participants may not have had active breaks as instructed. In addition, we did not monitor the daily occupational sitting behavior of participants in the control group, who received a placebo seat pad made of polypropylene foam to sit on. Thus, a comparison of occupational sitting behaviors between the intervention and control groups is not possible. These limitations may affect the internal validity of the present study. Future study should examine the efficacy of active breaks and postural shifts to prevent neck and low-back pain in those with poor habitual sitting behavior relative to the designated active breaks and postural shifts suggested by the apparatus to validate the present findings.

### Concluding remarks

A 3-arm, cluster-randomized controlled trial was conducted in a convenience sample of healthy office workers drawn from six organisations located in Bangkok, Thailand, comprising mainly middle-aged females with ≥5 years working experience and high risk of neck and low-back pain. Our results suggest that the active break and postural shift interventions delivered by the custom-designed apparatus can effectively reduce new onset of neck and low-back pain in these office workers. However, neither the active break nor postural shift intervention decreased pain intensity and disability in those experiencing neck and low-back pain.

### Competing interests

The authors declare no conflicts of interest.
